# All insects matter: a review of 160 entomology cases from 1993 to 2007 in Switzerland—part I (Diptera)

**DOI:** 10.1093/jme/tjad164

**Published:** 2023-12-29

**Authors:** Jiri Hodecek, Luca Fumagalli, Pavel Jakubec

**Affiliations:** Swiss Human Institute of Forensic Taphonomy, University Centre of Legal Medicine, Lausanne, Switzerland; Musée Cantonal de Zoologie, Palais de Rumine, Lausanne, Switzerland; Swiss Human Institute of Forensic Taphonomy, University Centre of Legal Medicine, Lausanne, Switzerland; Laboratory for Conservation Biology, Department of Ecology and Evolution, Biophore, University of Lausanne, Lausanne, Switzerland; Department of Ecology, Faculty of Environmental Sciences, Czech University of Life Sciences Prague, Suchdol, Czech Republic

**Keywords:** real cases, forensic entomology, checklist, species composition, necrophagous Diptera

## Abstract

Necrophagous Diptera are the most important group of insects used for the purposes of forensic entomology. While the most utilized fly family in this context is the family Calliphoridae, there are several other families that can be of great importance during real-case investigations. This article analyzes the necrophagous flies of all families recorded from 160 real cases in Switzerland between 1993 and 2007. A total of 56 species belonging to 16 families was identified with Calliphoridae being the most dominant family (90.63% of all cases), followed by Muscidae (26.25%), Sarcophagidae (19.38%), Phoridae (14.38%), and Fanniidae (12.50%). For specimens that were difficult to identify morphologically, a new PCR primer has been specifically designed for the amplification of a short, informative COI barcode in degraded museum samples of forensically important Diptera taxa. The richest family in terms of species was the family Muscidae with 16 species. *Fannia fuscula* (Fallen) and *Fannia monilis* (Haliday) were recorded from human cadavers for the first time. The study highlights the importance of different fly families in forensic investigation, enhancing our comprehension of their prevalence and dispersion in real cases in Central Europe. The results pave the way for additional exploration, especially regarding the involvement of less frequently observed species in forensic entomology.

## Introduction

The primary aim of forensic entomology is to estimate the minimum postmortem interval (PMI_min_) (Amendt et al. 2007). For forensic pathologists, it is extremely difficult to precisely estimate the time of death when the death occurred more than 3 days ago (Campobasso a Introna, 2001). In such cases, insects can become the best tool for PMI_min_ estimation due to the rapid colonization of cadavers by necrophagous flies (Amendt et al. 2011). The first necrophagous flies (usually from the family Calliphoridae) can arrive within minutes to hours after death and begin laying eggs (Amendt et al. 2011). As fly development is almost linearly dependent on temperature, forensic entomologists can calculate the day of fly oviposition after obtaining the temperature profile from the crime scene (Wells and Lamotte 2010). For this reason, forensic entomology is a well-established branch of forensic sciences, frequently used in criminal investigations worldwide (Byrd and Tomberlin, 2020).

Forensically important insect species colonize cadavers continuously in a series of overlapping occurrences (Matuszewski et al. 2011). When we encounter a body with the first colonizing species of necrophagous flies still present, and we have correctly reconstructed the temperature the insects were exposed to during their development, PMI_min_ estimation is quite precise as long as we correctly identify the oldest developmental stage of the fly that colonized the body (Byrd and Tomberlin, 2020). However, for cadavers in an advanced or mummified stage of decomposition, we need to use a different approach, and the PMI_min_ is estimated based on species composition (Amendt et al. 2011, Byrd and Tomberlin, 2020, Pittner et al. 2020). For this, we need to correctly identify the species of the entire insect community colonizing the cadaver while being aware of the ecology of the present species (Amendt et al. 2011). All present species might then be potentially forensically important, due to their specific spatiotemporal distribution, seasonality, and ecological requirements. “Forensically important” species are meant species that can help in any way to an ongoing criminal investigation. In the majority of cases, the analysis of the first colonizers (usually the flies of the family Calliphoridae) is sufficient for PMI_min_ estimation, and that is why most articles in forensic entomology focus on making it even more precise. In addition, the first colonizers of necrophagous insects received the most attention. Nevertheless, the later colonizers are of great importance when dealing with bodies in advanced decay. Unfortunately, many species, which could be used for the PMI_min_ estimation in such cases are hard to identify and/ or lack developmental data. We also often don’t know anything about their biology and ecological requirements. As the research should go hand in hand with casework to provide solutions for the questions casework poses, we need to focus more on the casework to know which questions are the most important ones (Hall 2021). Real-case data are sometimes published as “case reports” in scientific journals and such publications have enormous value in (i) describing unusual cases; (ii) providing a comparison of similar cases; (iii) identifying local fauna, and (iv) discussing the application and validation of methods (Hall 2021). Although, there is recently an increasing number of case reports published, drawing meaningful conclusions can usually be based only on more complex datasets (Hu et al. 2023). Unfortunately, such datasets are extremely scarce, and successional data from human remains are often missing completely.

To date, several published commented checklists of forensically important insects (of at least 20 cases including species of later phases of decomposition) from human carcasses exist: Carvalho et al. (2000) determined the most important insect species of forensic importance based on the abundance of necrophagous insects collected from pigs and humans in Southeastern Brazil. Sukontason et al. (2007) published a list of insect species found on 30 human bodies during the years 2000–2006 in Thailand. Lefebvre and Gaudry (2009) analyzed 356 expert works of the Department of Forensic Entomology of the French Gendarmerie, which were conducted on the whole French territory during 1992–2003. Dekeirscchieter et al. (2013) summarized 36 years of practice of Dr. Leclercq, which includes 132 real-case scenarios and 100 insect species. Farrell et al. (2015) reviewed necrophagous insects from 20 cases of human remains and 82 nonhuman vertebrate remains from Southeast Queensland, Australia. Sanford (2017) analyzed 203 forensic entomology cases from 2013 to 2016 in Houston, TX, USA. Lutz et al. (2021) wrote an extensive study of all insect species collected from 279 cases in Germany during the years 2001–2019 and Hu et al. (2023) then compiled 307 forensic entomology case reports from the literature of 1935–2022. In this manuscript, we present a large-scale dataset of all Diptera species found during 160 entomology cases in Switzerland over a 14-year period from 1993 to 2007.

## Material and Methods

**Fig. 1.**
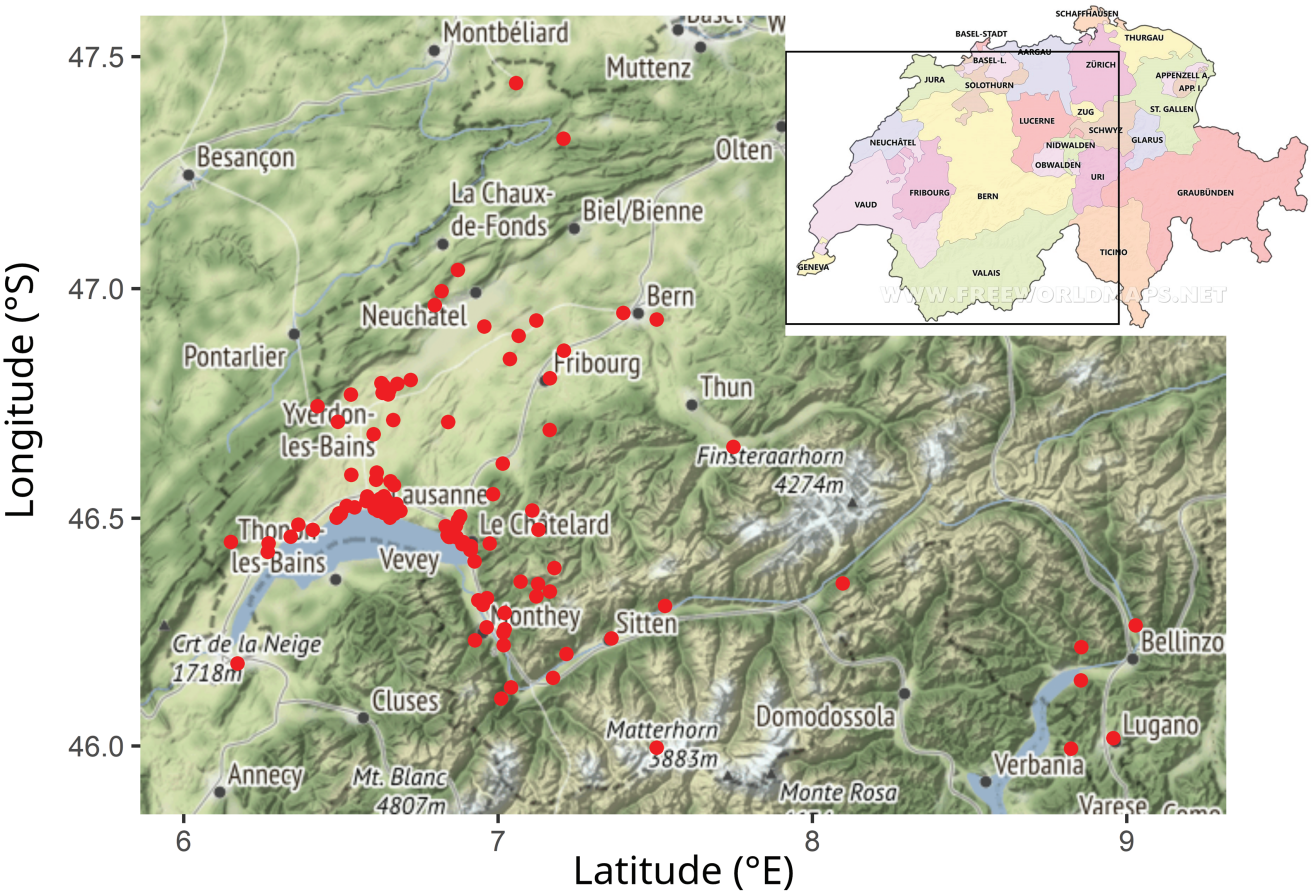
The map of real-case investigations in and around Lausanne during 1993–2007 (the map of Switzerland cantons downloaded from www.freeworldmaps.net).

The data come from 160 criminal investigations involving entomological expertise, which were conducted between the years 1993 and 2007 in and around Lausanne in Switzerland. The area contains cantons Vaud, Geneva, Fribourg, Neuchatel, Jura, Bern, Valais, and Ticino (Fig. 1). For the analyses, the information about the PMI_min_ and the discovery dates were used. Based on Lutz et al. (2021), the PMI_min_ was classified into 5 groups (1–7 days, >1–3 weeks, >3 weeks to 3 months, >3–6 months, and >6 months). The PMI_min_ was available for 140 cases. Unfortunately, the table we were working with lacked information on how the PMI_min_ of certain cases was calculated (i.e., we do not always know what exact species was used for the PMI_min_ estimation).

The insects were collected by Claude Wyss, who served with the police during the 1990s and early 2000s. The methods employed for collecting insect specimens are detailed in Cherix et al. (2012). Immature stages of necrophagous flies found on cadavers were raised in a controlled laboratory environment to adulthood, and later utilized for PMI_min_ calculations. All acquired insect samples were prepared and preserved in the Museum of Zoology in Lausanne. Identification of the blowflies was based on their terminalia employing a combination of identification keys and the reference collection at the Zoological Museum, University of Copenhagen. The blowflies were initially identified by C. Wyss, D. Cherix, and J. Faucherre. Between 2019 and 2020, Hodecek revised the material, working on rectifying and updating the database, which contained errors, outdated names, and unidentified specimens. The revision was made based on the actualized available keys (Szpila 2012—Calliphoridae; Szpila unpublished—Sarcophagidae + other families of forensic importance; Grzywacz unpublished—Muscidae and Fanniidae).

When morphological identification was difficult, we used a DNA barcoding approach. The DNA from 66 pinned museum specimens (single legs, whole bodies for small flies or pupae aged 16–30 years) was extracted with the QIAamp DNA Micro Kit (Qiagen, Hilden, Germany) in a laboratory restricted to forensic or low DNA-content analyses. Because the use of museum specimens to generate full-length barcodes is challenging due to potential DNA degradation, we amplified a short amplicon (136 bp) of the mitochondrial cytochrome *c* oxidase subunit I (COI) with primer type R3 (Hebert et al. 2013) and a newly designed primer with greater specificity to forensically relevant Diptera families such as Sarcophagidae, Phoridae, and Piophilidae (L_Sarc: 5ʹ-CCTATTATAATTGGGGGATTTGG-3ʹ). The PCR reactions were performed in 25 µL. The mixture contained 1× PCR Gold Buffer (Thermo Fisher Scientific, USA), 2 mM MgCl_2_, 0.2 mM of dNTPs, 0.2 mg/mL of bovine serum albumin (Roche Diagnostics, Basel, Switzerland), 0.5 µM of forward and reverse primers, 1 U of AmpliTaq Gold (Thermo Fischer Scientific, USA) and 3 µL of template DNA. PCR cycling conditions were 3 min denaturation at 95°C, followed by 50 cycles of 30 s at 95°C, 30 s at 50°C, and 1 min at 72°C, with a final elongation step of 5 min at 72°C. PCR products were purified using the QIAquick PCR Purification Kit (Qiagen) before Sanger sequencing in both directions at Microsynth AG (Balgach, Switzerland). The obtained COI sequences were aligned and blasted in GenBank, to relate them to reference sequences and identify them. To refine the identification in case of ambiguous (<100% identity) assignments, we also built a distance tree rooted with the homologous sequence of *Bradysia ocellaris* (Comstock) with Seaview 5.0.4 (BioNJ (Gascuel 1997), K2P distances, 1,000 bootstrap replicates (Galtier et al. 1996, Gouy et al. 2010); and clustered the sequences with reference sequences of *Sarcophaga argyrostoma* (Robineau-Desvoidy) (accession nr. M679858); *Megaselia abdita* Schmitz (GU075399); *Megaselia scalaris* (Loew) (MT111889); *Megaselia rufipes* (Meigen) (MT472135); and *Megaselia lucifrons* (Schmitz) (MN672285) (data not shown). DNA haplotypes produced in this study are available on Dryad (https://doi.org/10.5061/dryad.0gb5mkm7p).

The statistic software R (R Core Team 2021) was used for the data restructuring and to create graphical outputs, while utilizing packages dplyr and ggplot2 (Wickham 2009, Wickham et al. 2023). The cases were separated based on the location of the body, where the cases that occurred in regularly inhabited buildings were considered as indoor and any other location such as natural environment, but also the interior of cars, shacks, or cabins were categorized as outdoor cases. Comparison of the species diversity among different habitats (indoor vs. outdoor) was done using the Wilcoxon rank-sum test with continuity correction. We further analyzed cases across meteorological seasons (Spring, Summer, Winter, and Autumn), where the cases were categorized accordingly by the discovery date. The original PMI_min_ values were for some cases calculated in months; these were for our purposes transformed to days by multiplying the original value by 30 to allow comparison. Also, some PMI_min_ were not calculated in the original database; these were treated as NA values and do not appear in the accompanying graphs. On the contrary, some of the original PMI_min_ values were not based on breeding of insect material and it is unknown if these were derived from species composition and succession, witness testimonies, or other methods, however, we treat them as informative values.

## Results and Discussion

**Fig. 2.**
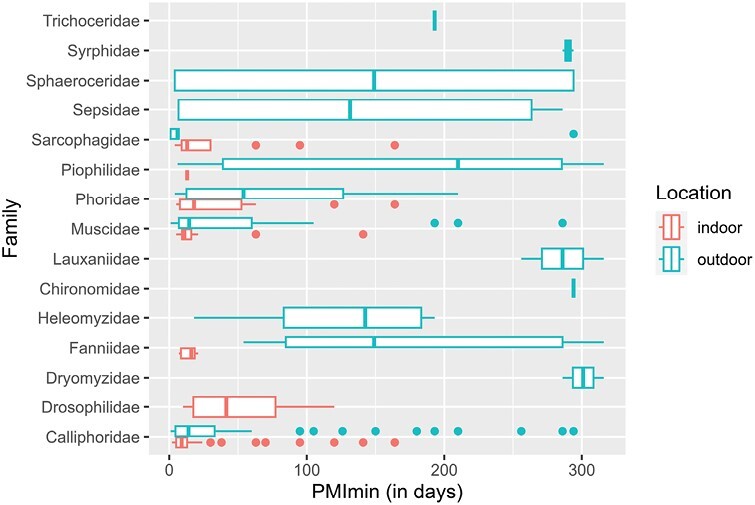
Boxplots of the distribution of the PMI_min_ (in days) across different families of Diptera with regards to the location where the body was discovered (indoor/outdoor habitat). The vertical lines within the boxes represent median values; the whiskers represent the values with the 1.5 interquartile ranges; the blue and red dots are outliers. Detailed figures for species of the major families can be found at Supplementary Fig. S1.

**Fig. 3.**
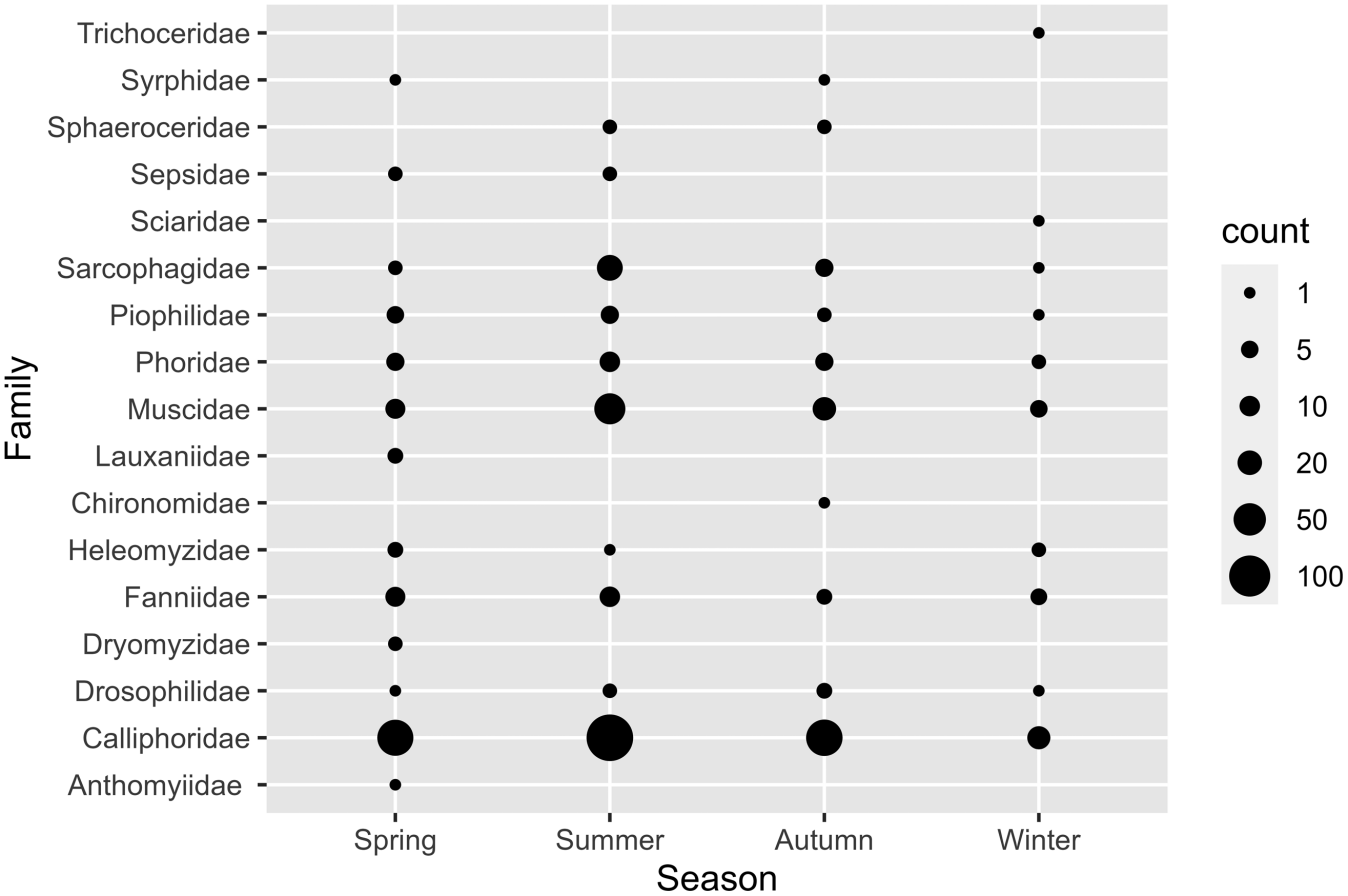
The occurrence of the collected Diptera families at different seasons. The size of the dot represents the number of records for given taxa and season. Detailed figures for species of the major families can be found at Supplementary Fig. S2.

A total of 56 species of Diptera belonging to 16 families were identified. Of the 66 specimens genetically analyzed, we were able to obtain DNA sequence information for 61 of them (94%) and correctly delineate with this short barcode the sequences down to the species or genus level. Necrophagous flies were present in 158 out of 160 real-case investigations (i.e., 98.75%). Out of the 158 cases, 92 (i.e., 58%) were located indoor (Fig. 2). The most dominant family was the family Calliphoridae, which was present in 145 cases (90.63%). The following families were: Muscidae in 42 cases (26.25%), Sarcophagidae in 31 cases (19.38%), Phoridae in 23 cases (14.38%), and Fanniidae in 20 cases (12.50%). The most species-rich family was the family Muscidae with 16 species (Table 1). Interestingly, most families were recorded in Spring (13) followed by Summer, Winter, and Autumn (all 10 families each) (Fig. 3).

**Table 1.** Diptera families sampled from real cases in Switzerland in between 1993 and 2007

Family	No. of species	No. of cases	% of cases
Calliphoridae	10	145	90.63
Muscidae	17	42	26.25
Sarcophagidae	5	31	19.38
Phoridae	2	23	14.38
Fanniidae	4	20	12.50
Piophilidae	2	14	8.75
Drosophilidae	1	7	4.38
Heleomyzidae	1	6	3.75
Sepsidae	1	4	2.50
Lauxaniidae	1	3	1.88
Dryomyzidae	1	2	1.25
Syrphidae	1	2	1.25
Anthomyiidae	1	1	0.63
Chironomidae	1	1	0.63
Sphaeroceridae	1	1	0.63
Trichoceridae	1	1	0.63
Total	56	160	

Comparison of outdoor and indoor cases in terms of species diversity encountered during each case was inconclusive (Wilcoxon rank-sum test: *W* = 2616, *P*  = 0.174). The maximum number of recorded dipteran species was 15 at the outdoor case closely followed by 13 species recorded at the indoor case. Most bodies were however infested by 1 (outdoor = 26.15%, indoor = 30.43%) or 2 (outdoor = 20.00%, indoor = 26.09%) species. Median number of species at different habitats slightly differed in favor of outdoor cases (outdoor = 3, indoor = 2). These results are in concordance with the results of Lutz et al. (2021), where monocolonization occurred at 30.11% and 2 species were found at 24.38% of the cases. However, the highest number of species (Diptera and Coleoptera together) recorded by Lutz et al. (2021) was only 10 species (in our analysis mentioned above only Diptera were included). The species, that seemed to prefer to colonize a body alone the most, were *Calliphora vicina* Robineau-Desvoidy (26% of all cases) and *Calliphora vomitoria* (Linnaeus) (16.67% of all cases).

### Calliphoridae

Blowflies are the most common colonizers of cadavers, and they are used for the calculation of the PMI_min_ by forensic entomologists due to their early arrival at the body (Amendt et al. 2011, Byrd and Tomberlin 2020). Since a separate article focused solely on Calliphoridae from the dataset was already published (Hodecek and Jakubec 2022), we will mention just a short summary here.

The most frequently occurring species in our dataset were *C. vicina* (100 cases—68.97%), followed by *Lucilia sericata* (Meigen) (44 cases—30.34%), *C. vomitoria* (42 cases—28.97%) and *Lucilia caesar* (Linnaeus) (39 cases—26.9%). All these 4 species are very important forensic indicators not only in Switzerland but also in the whole Palearctic region (Szpila 2012). However, there is a difference in their respective abundance in comparison with other available European datasets from real cases (Fremdt and Amendt 2014, Bernhardt et al. 2018, Feddern et al. 2018, Lutz et al. 2021). While in other European datasets *L. sericata* is usually the most dominant species, in Switzerland it was by far *C. vicina*. Also, the position of other species is slightly different, and the reason might be the different local topography (Hodecek and Jakubec 2022). The distribution of blowflies from this dataset, their affinity to different types of biotopes, altitude, and season can be found in Hodecek and Jakubec (2022). The occurrence of the calliphorids based on the PMI_min_ and their seasonal distribution can be found in Supplementary Fig. S1.

### Sarcophagidae

Flesh flies is a family of hard-to-identify flies with promising potential in forensic entomology. While adult males can be identified mostly by their genitalia, females are almost impossible to distinguish morphologically (Szpila et al. 2015). Therefore, there are attempts to ease the identification problem by different approaches (i.e., by DNA barcoding: Meiklejohn et al. 2011; or newly by wing morphometrics: Szpila et al. 2022). Although there are a lot of species, that can be potentially attracted by a carrion, there are only a few that have been recorded to develop in it (Szpila et al. 2015). Similarly, to the family Calliphoridae, records of flesh flies from the dataset were already extensively studied in a separate manuscript (Cherix et al. 2012), thus we are bringing just a shortened summary.

In Central Europe, the most dominant species in real-case investigations is *S. argyrostoma* (Szpila et al. 2015, Lutz et al. 2021), which was the case also in our dataset (26 cases, 16.25%). Another forensically important species is *Sarcophaga caerulescens* Zetterstedt, although it seems to be more common on pig carrions (Szpila et al. 2015) rather than on humans (Pohjoismäki et al. 2010). *Sarcophaga caerulescens* was the only other species, which has been recorded from multiple cases in our dataset (i.e., 3; 1.88%), while the other 2 species (*Sarcophaga africa* [Wiedemann] and *Sarcophaga similis* [Meade]) were recorded just once. Cherix et al. (2012) mentioned also *Sarcophaga dux* Thomson, however, the single specimen caught was a female (which is very hard to identify) and the initial identification was thus incorrect. The specimen was reexamined by Dr. Daniel Whitmore from the Stuttgart State Museum of Natural History, who confirmed that it is a species belonging to the *Sarcophaga carnaria* group. More information about the occurrence and importance of Sarcophagidae as forensic indicators in Switzerland can be found by Cherix et al. (2012). The occurrence of the sarcophagids based on the PMI_min_ and their seasonal distribution can be found in Supplmentary Fig. S2.

### Muscidae

Muscidae is a dipteran family rich in potentially forensically relevant species (Grzywacz et al. 2017). Unfortunately, the relevance of their usage in real-case investigations is momentarily lowered by the difficulty of their identification and lack of developmental data. The same way as for Sarcophagidae, this motivates for finding of new ways of species identifications based on DNA barcoding (Ren et al. 2018) or even wing measurements (Grzywacz, Ogiela, et al. 2017). Muscids are very diverse group with species of forensic interest being found in sunny or shaded places; wet or dry habitats; they are attracted by corpses indoor as well as outdoor in both—early and advanced states of decomposition (Grzywacz et al. 2017) (for our data see Supplementary Fig. S1). They usually arrive after Calliphoridae and Sarcophagidae, but there are cases where Muscidae are the only present flies (probably due to limited access) (Smith 1986).

In our dataset, Muscidae was the richest family with 16 species. The most abundant was *Hydrotaea ignava* (Harris) present at 19 cases (11.88%) followed by *Hydrotaea dentipes* (Fabricius) in 13 cases (8.13%) and *Hydrotaea aenescens* (Wiedemann) in 7 cases (4.38%). In Lutz et al. (2021), these 3 species of the genus *Hydrotaea* were also amongst the most abundant muscids exactly in the same order as in our dataset (*H. ignava* in 8 cases, *H. dentipes* in 6 cases, and *H. aenescens* in 3 cases, respectively). However, in their dataset, the most dominant muscid was *Musca domestica* Linnaeus (10 cases in Germany vs. up to 3 cases in Switzerland). The biology of *H. ignava* and *H. dentipes* is very similar and these 2 species often occur together (Skidmore 1985). This could also be confirmed in our dataset as *H. dentipes* was found altogether with *H. ignava* in 6 out of 13 cases. The larvae of *Hydrotaea* are often predatory feeding on larvae of other necrophagous flies such as *Musca* or even *Calliphora*. It was recorded that the coexistence of *H. ignava* and *M. domestica* can lead to a significant number reduction of *M. domestica* larvae (Skidmore 1985). *Hydrotaea ignava* was also the first species of its genus to be sequenced for its complete mitochondrial genome (Karagozlu et al. 2017) followed by *H. dentipes* (Yan et al. 2019). Due to their frequent occurrence in human cadavers, there is an increasing need for the breeding data of these species. From the other genera of this family, we can mention the species of the genus *Muscina*, which were present in 5 (*Muscina prolapsa* [Harris]), 4 (*Muscina stabulans* [Fallen]), and 3 cases (*Muscina pascuorum* [Meigen]), respectively. Muscids of this genus also often feed on other dipteran larvae and can cause a decrease in their numbers, which always has to be considered during forensic investigations, i.e., when we encounter a body with both species present, we have to be aware that the oldest developmental larval stage of *M. domestica* could be missing due to predatory activities of *Muscina* sp. and thus warn the investigators that our estimation might be undervalued (Skidmore, 1985, Grzywacz, Hall, et al. 2017).

In our dataset, muscids were accompanied by the presence of calliphorids in 40 out of 42 cases. In one case, there was only *H. capensis* altogether with *Fannia scalaris* (Fabricius) found outside in January and in the other case, there was *H. aenescens* with *M. scalaris* in indoor case in October.

### Phoridae

Phoridae or “the scuttle flies” are forensically important family of small flies often found in indoor cases with limited access to the blowflies (e.g., closed windows or even sealed plastic bag) (Greenberg and Wells 1998, Manlove and Disney 2008). This increases their potential value as forensic indicators, as they can be the only necrophagous insects at the scene (Reibe and Madea, 2010, Zuha and Omar, 2014). They were found in 23 cases of this study (14.38%) of which 16 (69.57%) were in an enclosed environment (apartment, car). Their affinity to indoor cases is in concordance with the results of other authors (e.g., 80% in Lutz et al. 2021). Phorids can colonize cadavers a few days after the death occurred (*Megaselia* sp.), but also in a very late phase of decomposition (*Conicera* sp.) (Martín-Vega et al. 2011) (for our data please see Supplementary Fig. S1).

We identified 4 species of phorids in the dataset. We used DNA barcoding to identify most specimens from this family. However, due to the potential DNA degradation of some of the samples and also some missing specimens, we were not able to identify all of the insects from the recorded collection. The results showed us that the most abundant species were *M. scalaris* and *M. abdita*, both recorded from 6 cases (3.75%), followed by *M. rufipes* in 4 cases (2.5%) and *Conicera* sp. in 2 cases (1.25%). This species composition slightly differs from the findings of Lutz et al. 2021, who found 68% of their identified phorids to be *M. scalaris*, followed by only one record of *M. abdita* and *M. rufipes*. The species richness was then higher in the dataset of Lutz et al. 2021 (6 species vs. 4 species in our dataset). *Megaselia scalaris* is one of the most common necrophagous species, which is often found on human bodies (Reibe and Madea 2010, Zuha and Omar 2014, Lutz et al. 2021). It is a cosmopolitan species with a humpbacked appearance, similar to the rest of the hundreds of species belonging to the same genus. It has been used as an important forensic indicator in numerous cases and it has been the most studied species of forensically important phorids (Greenberg and Wells, 1998, Reibe and Madea, 2010, Zuha and Omar 2014, Zhong et al. 2016). *Megaselia abdita* and *M. rufipes* have similar ecological requirements as *M. scalaris*, they are all cold-tolerating species and these 2 species in particular were recorded on human bodies during winter (Disney and Manlove 2005, Manlove and Disney 2008).

In 3 out of 23 cases in our dataset, there was no calliphorid and/ or sarcophagid present altogether with the phorid. Apart of the case, where *M. scalaris* was present altogether with *H. aenescens*, which was already mentioned earlier, there were 2 other indoor cases. In one of them there was only *M. rufipes* present on the body, while in the other one, there was only *M. abdita*. Any of these 3 species could thus be our only tool to use for the PMI_min_ estimation in certain indoor cases.

### Fanniidae

There were 20 cases (12.5%) with flies from the family Fanniidae in our dataset. Fanniidae is a family of rather small flies, which can be found in both—indoor and outdoor cases. Their usefulness in real-case investigations is unfortunately hindered by their fairly difficult morphological identification and lack of developmental data. They are attracted by the bodies in early, moist stages or advanced stages of decomposition (Grzywacz et al. 2017) (see Supplementary Fig. S1). Similarly, to muscids and phorids, they also sometimes colonize bodies in places inaccessible to bigger flies due to their smaller body size. They are also known to inhabit buried remains (Bourel et al. 2004).

A total of 5 species were recorded amongst Fanniidae. The most frequent was *F. scalaris* present in 11 cases (6.88%) followed by *Fannia manicata* (Meigen) in 8 cases (5%). The other 3 species (*Fannia canicularis* [Linnaeus], *Fannia monilis*, and *Fannia fuscula*) were found only once. In our cases, they were found more frequently outdoors (65%). There were 3 outdoor cases with live larvae during the cold season (25th November–7th January), which is an interesting observation as they are rarely found in cold months. However, the PMI_min_ in those cases were more than 3 months, which means that the female laid eggs during warmer months. *Fannia scalaris* and *F. manicata* were the most abundant species in Germany (Lutz et al. 2021). Out of these 5 species, the only species for which we have developmental data (Grzywacz, 2019) and sequenced mitochondrial genome (Ge et al. 2022) is *F. canicularis*. It would be strategic to do similar work on *F. scalaris* and/or *F. manicata* as they seem to be important forensic indicators in Central Europe. Until now, *F. monilis* and *F. fuscula* have been recorded only from animal carrions (Rozkošný et al. 1997, Hwang and Turner, 2005, Grzywacz and Prado Castro 2012), which makes this their first record on human bodies. However, only *F. monilis* was found in larval stadium. *Fannia fuscula* was caught only as an adult visiting the body (the information about the developmental stage of collected insects for the rest of the taxa can be found in Table 2).

**Table 2.** Table depicting the number of cases with adult vs. immature developmental stages present. The column “Adults” shows the number of cases with only adults present, while the column “Immature” shows the number of cases with any other developmental stage of the collected taxa. “N/A” is the number of cases with no available data, “No. of cases” is the total number of cases for each taxon, and “% of cases” is the percentage representation of the taxon. The species marked in bold are new records found on human bodies in Switzerland

Family	Species	Adults	Immature	N/A	No. of cases	% of cases
Calliphoridae	*Calliphora vicina*	16	81	3	100	62.50
	*Calliphora vomitoria*	7	34	1	42	26.25
	*Lucilia sericata*	7	35	2	44	27.50
	*Lucilia caesar*	16	23	0	39	24.38
	*Lucilia illustris*	13	6	3	22	13.75
	*Protophormia terraenovae*	9	10	2	21	13.13
	*Chrysomya albiceps*	3	6	4	13	8.13
	*Phormia regina*	2	4	1	7	4.38
	***Cynomya muortorum***	0	2	0	2	1.25
	*Lucilia ampullacea*	1	0	0	1	0.63
	*Lucilia* sp.	1	0	2	3	1.88
	Calliphoridae sp.	0	2	0	2	1.25
Muscidae	*Hydrotaea ignava*	10	7	1	18	11.25
	*Hydrotaea dentipes*	6	7	0	13	8.13
	*Hydrotaea aenescens*	4	2	1	7	4.38
	*Muscina prolapsa*	4	1	0	5	3.13
	*Hydrotaea capensis*	2	2	0	4	2.50
	***Hydrotaea irritans***	2	2	0	4	2.50
	***Muscina stabulans***	3	1	0	4	2.50
	***Hydrotaea similis***	3	0	0	3	1.88
	***Muscina pascuorum***	3	0	0	3	1.88
	***Muscina levida***	2	0	0	2	1.25
	***Hydrotaea pilipes***	0	1	0	1	0.63
	*Musca autumnalis*	0	1	0	1	0.63
	*Musca domestica*	1	0	0	1	0.63
	***Polietes lardarius***	1	0	0	1	0.63
	*Hydrotaea* sp.	2	1	0	3	1.88
	*Musca* sp.	2	0	0	2	1.25
	*Muscina* sp.	1	0	0	1	0.63
	*Thricops* sp.	1	0	0	1	0.63
	Stomoxyini	0	1	0	1	0.63
Sarcophagidae	*Sarcophaga argyrostoma*	4	21	1	26	16.25
	*Sarcophaga caerulescens*	1	2	0	3	1.88
	*Sarcophaga africa*	0	1	0	1	0.63
	*Sarcophaga similis*	0	1	0	1	0.63
	*Sarcophaga* sp.	1	0	1	2	1.25
Phoridae	***Megaselia abdita***	0	5	1	6	3.75
	*Megaselia scalaris*	0	6	0	6	3.75
	***Megaselia rufipes***	1	3	0	4	2.50
	*Megaselia* sp.	4	2	0	6	3.75
	*Conicera* sp.	1	1	0	2	1.25
Fanniidae	*Fannia scalaris*	3	6	2	11	6.88
	*Fannia manicata*	1	6	1	8	5.00
	*Fannia canicularis*	0	0	1	1	0.63
	***Fannia fuscula***	0	1	0	1	0.63
	***Fannia monilis***	1	0	0	1	0.63
	*Fannia* sp.	2	2	0	4	2.50
Piophilidae	*Stearibia nigriceps*	2	4	0	6	3.75
	***Allopiophila vulgaris***	1	2	0	3	1.88
	*Piophila casei*	0	3	0	3	1.88
	***Liopiophila varipes***	1	0	0	1	0.63
	*Piophila* sp.	0	1	0	1	0.63
Drosophilidae	***Drosophila funebris***	5	0	0	5	3.13
	Drosophilidae sp.	0	2	0	2	1.25
Heleomyzidae	***Neoleria inscripta***	1	1	0	2	1.25
	*Heleomyzidae* sp.	2	2	0	4	2.50
Sepsidae	Sepsidae sp.	2	2	0	4	2.50
Lauxaniidae	*Lauxaniidae* sp.	0	3	0	3	1.88
Sphaeroceridae	***Leptocera caenosa***	1	1	0	2	1.25
	***Spelobia luteilabris***	1	0	0	1	0.63
	***Telomerina flavipes***	0	0	1	1	0.63
Dryomyzidae	Dryomyzidae sp.	0	2	0	2	1.25
Syrphidae	***Syritta pipiens***	1	1	0	2	1.25
Anthomyiidae	Anthomyiidae sp.	1	0	0	1	0.63
Chironomidae	*Tricocera* sp.	1	0	0	1	0.63
Sciaridae	***Bradysia tilicola***	0	0	1	1	0.63
Trichoceridae	Trichoceridae sp.	1	0	0	1	0.63

In our dataset, there were several cases of fanniids not being accompanied by calliphorids or sarcophagids, which underline their importance for forensic entomology and PMI_min_ estimation. In 2 outdoor cases, fanniids were sharing the carrion only with piophilids (*F. scalaris* + *Piophila casei* (Linnaeus); unidentified species of *Fannia* + *Allopiophila vulgaris* [Fallen]), in another outdoor case, there were larvae of *F. scalaris* and *H. capensis* (already mentioned earlier) and in one indoor case, there were just *F. scalaris* and *F. canicularis* present.

### Piophilidae and the Rest of the Dipteran Families

The most abundant family of the rest of the present families was the family Piophilidae (14 cases, 8.75%). Although this family is quite small (~80 species), some species can be important forensic indicators (López-García et al. 2020). They usually colonize the cadaver with a delay of several weeks and prefer outdoor cases (Martín-Vega 2011, Matuszewski and Mądra-Bielewicz 2019) (see Supplementary Fig. S1). In our dataset, they were found indoors only in 1 out of the 14 cases, which supports this hypothesis. The overall dominance of indoor cases in real-case investigations might be the reason for the lower frequency of encountering these flies during forensic investigations. However, this does not decrease their importance for certain outdoor cases, where they can play an important role during the investigation (Martín-Vega 2011).

The identification of the specimens from this family was partially done by DNA barcoding and we identified 4 species in total. The most common species in our dataset was *Stearibia nigriceps* (Meigen) (6 cases, 3.75%), which was the most frequent piophilid in the dataset of Lutz et al. (2021). *Stearibia nigriceps* is usually associated with mummified and skeletonized corpses with PMI_min_ of 6 weeks to 9 months (Martín-Vega 2011). In our dataset, the PMI_min_ was also mostly in this range, however, there was 1 case, where the larvae of *S. nigriceps* were found in a case with PMI_min_ = 13 days. Another common piophilid often registered in human corpses is *P. casei* (Martín-Vega 2011). In fact, this species was for a long time considered as the only species from this family with some forensic relevancy (Martín-Vega 2011). We report 3 cases (1.88 %) of *P. casei* all with very long PMI_min_ (>9 months). On the contrary, *A. vulgaris* is not commonly reported from human corpses and we report it from 3 cases (1.88%) as well. Lastly, there was one case with *Liopiophila varipes* (Meigen), another rather uncommon species recorded from human bodies.

There were 3 cases, with juvenile stages of piophilids, while none of calliphorids and/or sarcophagids. Two of them were already mentioned earlier with *P. casei* and *A. vulgaris* accompanying species from the family Fanniidae. The last one was an outdoor case from August where only *P. casei* was present.

The rest of the dipteran families/species encountered during investigations in Switzerland is of a very little forensic importance and can be found in Table 2.

## Conclusion

The study provides a comprehensive description of the species composition of Diptera in forensic cases in Switzerland. It underscores the importance of various fly families in forensic investigations and contributes to the understanding of their occurrence and distribution in real-case scenarios. In addition, we have developed a short informative COI barcode, effective for DNA barcoding of pinned, old, and potentially degraded Diptera museum samples of forensic interest. Among the 160 real-case investigations, there were 56 species identified including new records of *F. fuscula* and *F. monilis* from human corpses. The research also opens new avenues for further investigation, particularly concerning the role of less common species in forensic entomology. After a thorough comparison of our dataset with the very similar dataset of Lutz et al. (2021), we noticed the difference in the recorded Diptera taxa (37 taxa in 279 cases in Lutz et al. 2021 vs. 56 taxa in 160 cases in our dataset) and concluded the importance of the sampling effort. While the insects in our dataset were collected by an entomologist, who collected them mostly at the crime scene, the insects in Lutz et al. (2021) were partially collected by other forensic personnel and often only during the autopsies (Lutz et al. 2021). This can lead to a significant under-sampling (Hall 2021, Matuszewski 2021). As it is clear from our dataset, there are some cases, where the whole entomology-related forensic work can depend on a species, which lacks developmental data and other related information in the literature. Our article tried to pinpoint such species for each family based on their appearance in real-case scenarios in Switzerland. An example of such cases from our dataset could be an indoor case with only *M. abdita* present; another indoor case with only *M. rufipes* present; an indoor case, where the only 2 species were *F. canicularis* and *F. scalaris*; an outdoor case with *A. vulgaris* and unidentified species of *Fannia*; an outdoor case where only *P. casei* was present and many other cases discussed earlier. These species should be focused on in further research as they seem to be of importance to forensic entomologists in Central Europe during their routine work. This dataset can also be used as a reference point for distribution and frequency of occurrence of all necrophagous flies in real-case scenarios in Europe.

## Supplementary Material

tjad164_suppl_Supplementary_Figures
