# Supplementary material for: All insects matter: a review of 160 entomology cases from 1993 to 2007 in Switzerland—part I (Diptera)
Source: J Med Entomol. 2023 Dec 29;61(2):400–9. doi: 10.1093/jme/tjad164 (PMC10936168; doi:10.1093/jme/tjad164)

Supplementary data.

**Figure S1.** Boxplots of the distribution of the PMI_min_ (in days) for the lowest determined taxonomical level across different families of Diptera with regards to the location where the body was discovered (indoor/outdoor habitat). The vertical lines within the boxes represent median values; the whiskers represent the values with the 1.5 interquartile ranges; the blue and red dots are outliers.


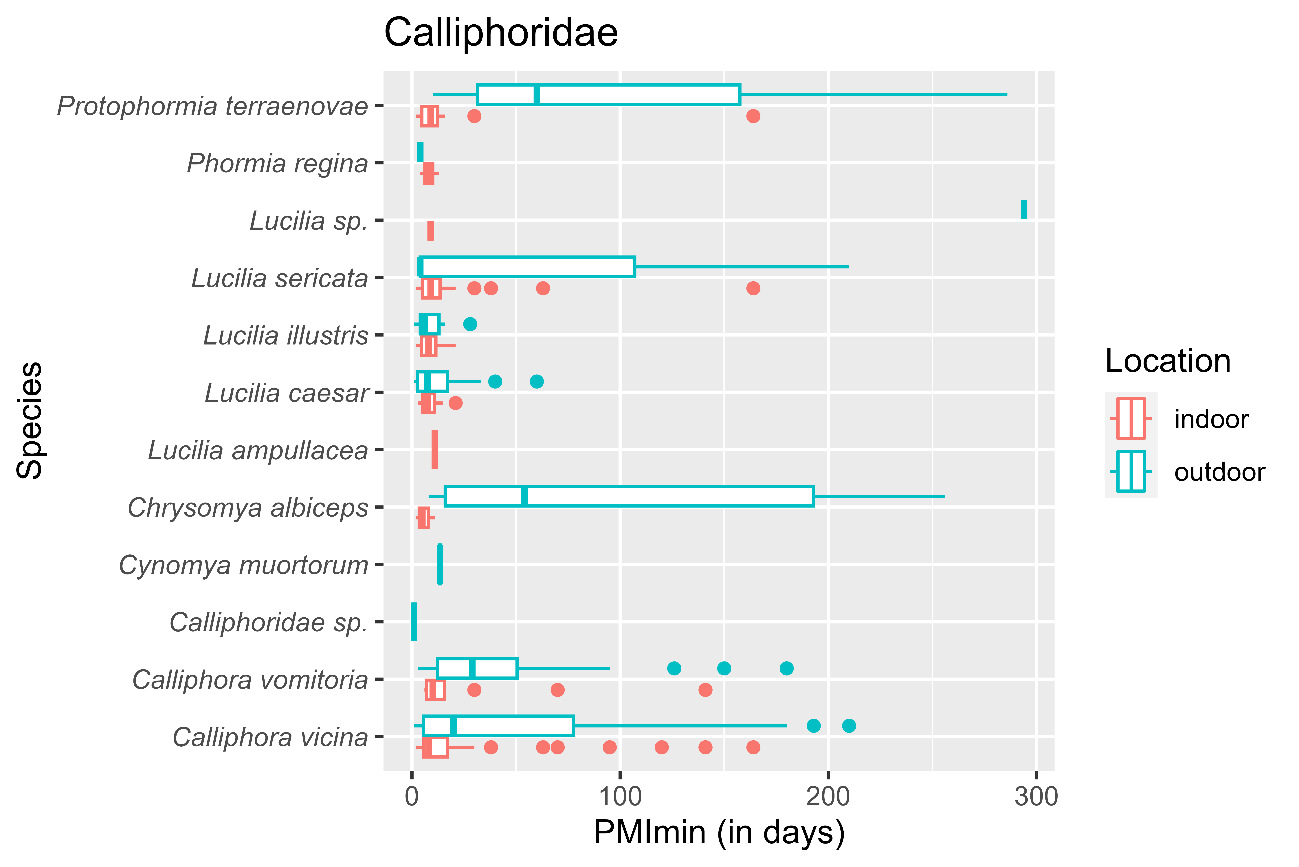

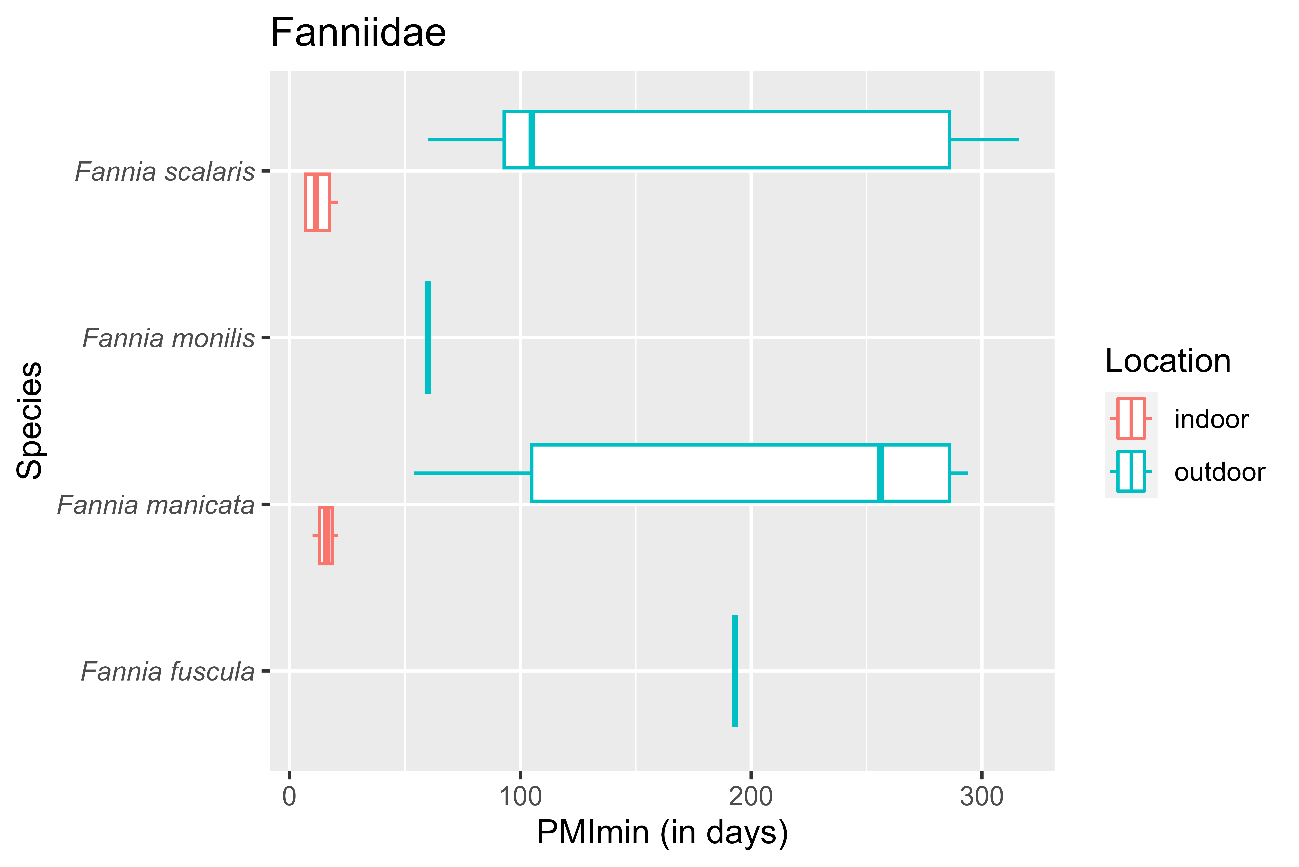

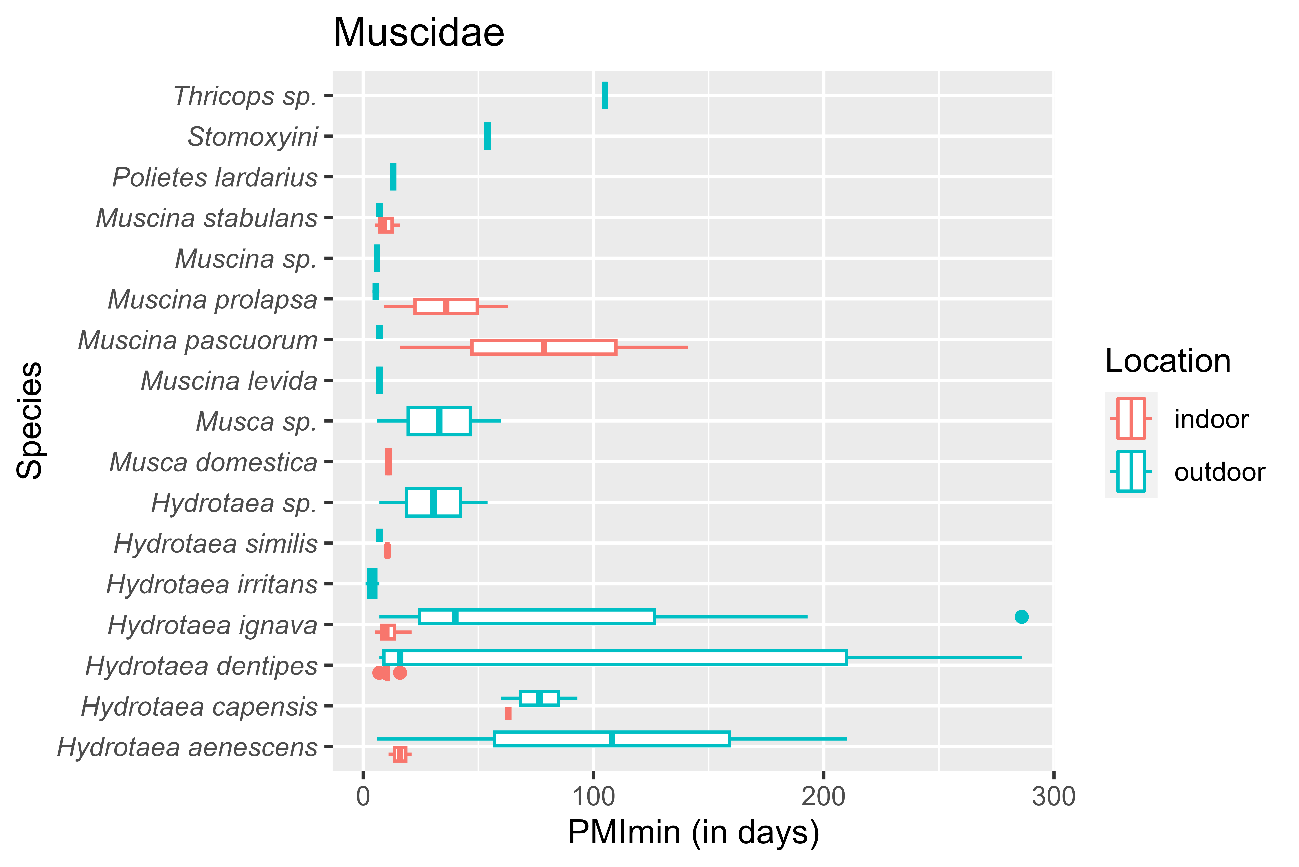

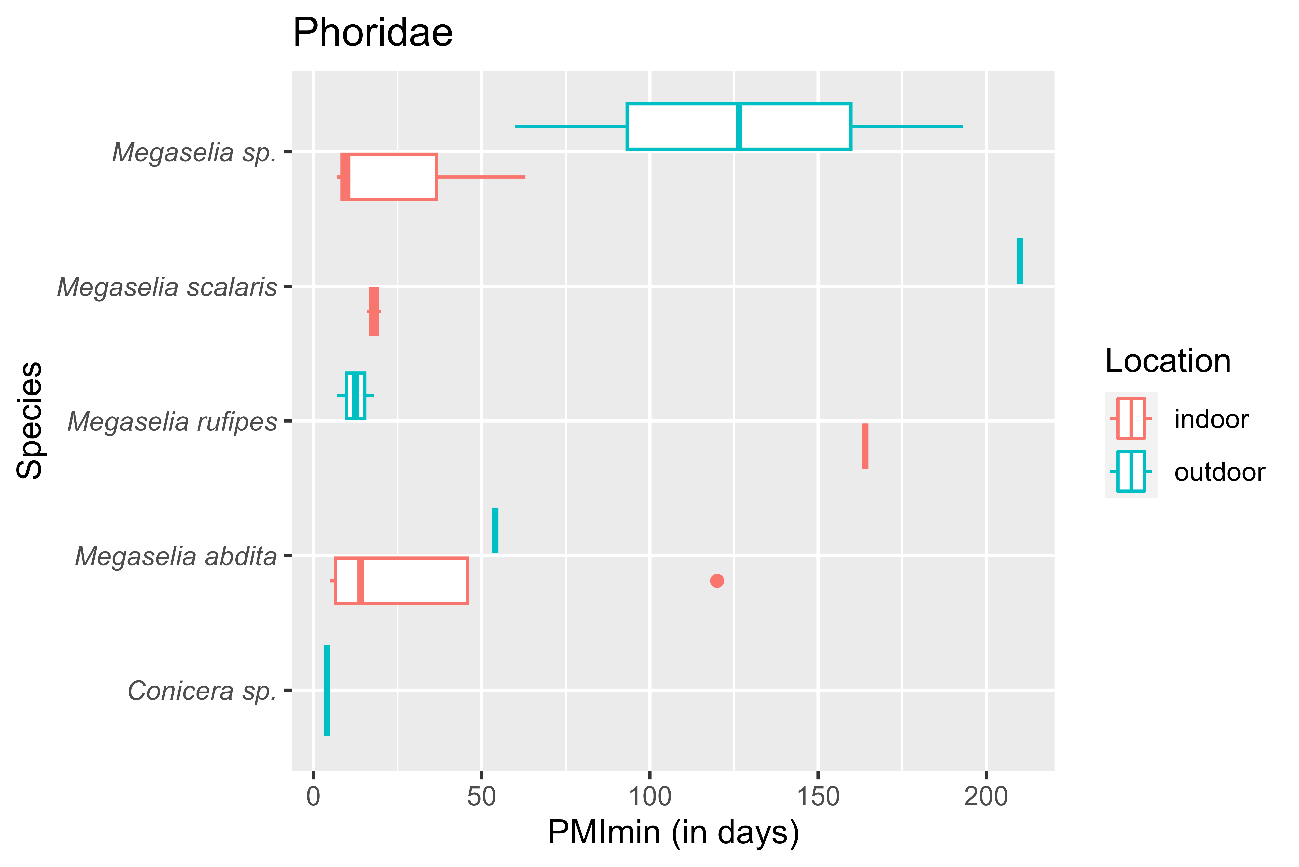

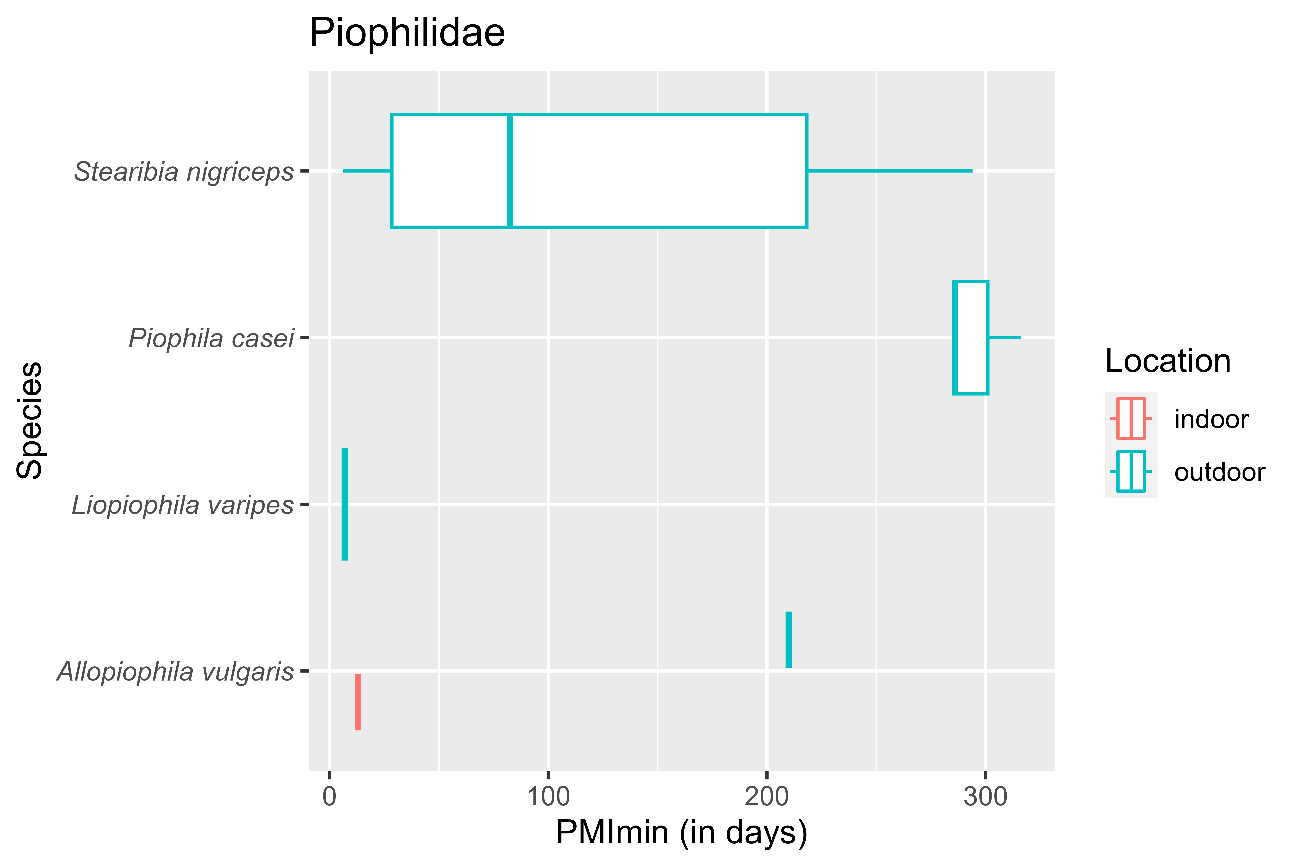

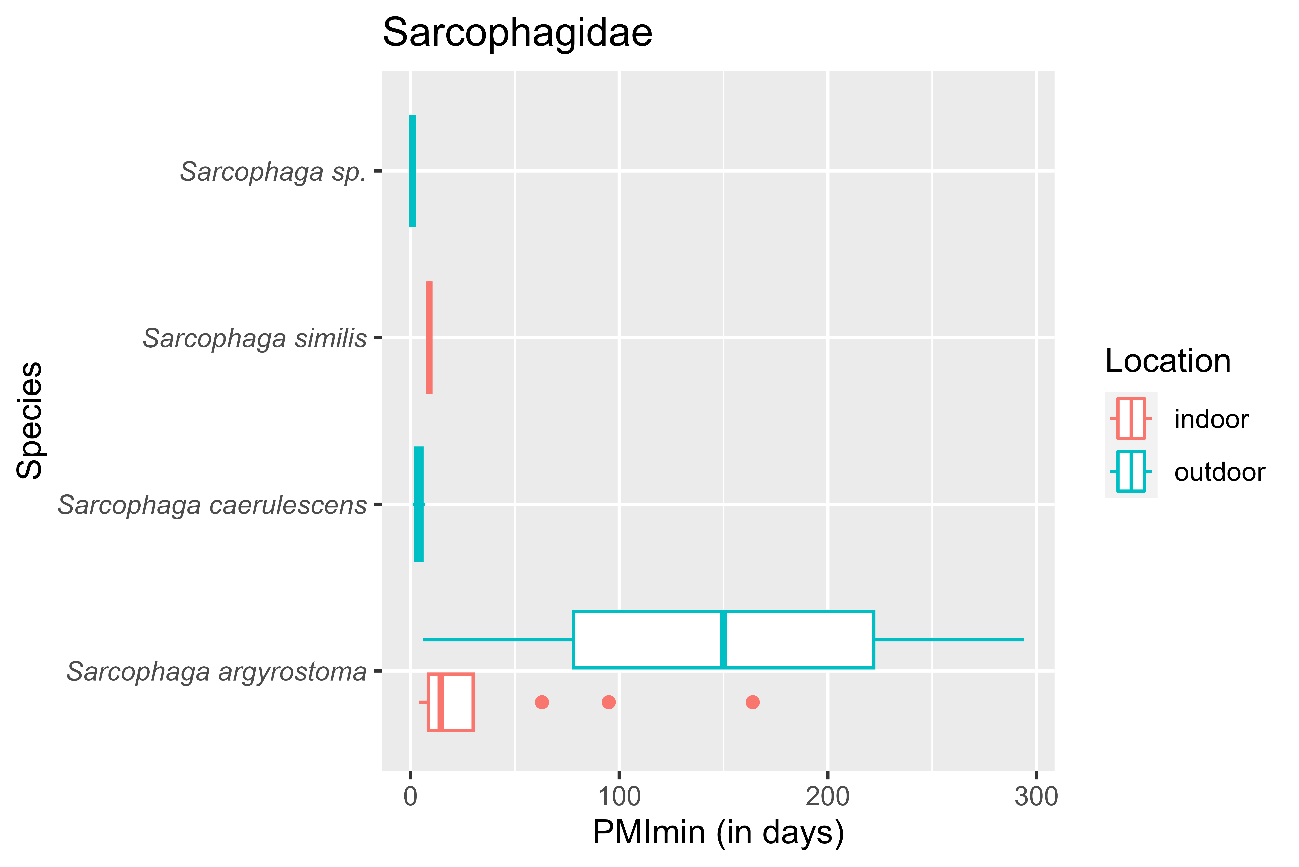


**Figure S2.** The occurrence of the collected species or the lowest determined taxonomical level of the Diptera families at different seasons. The size of the dot represents the number of records for given taxa and season.
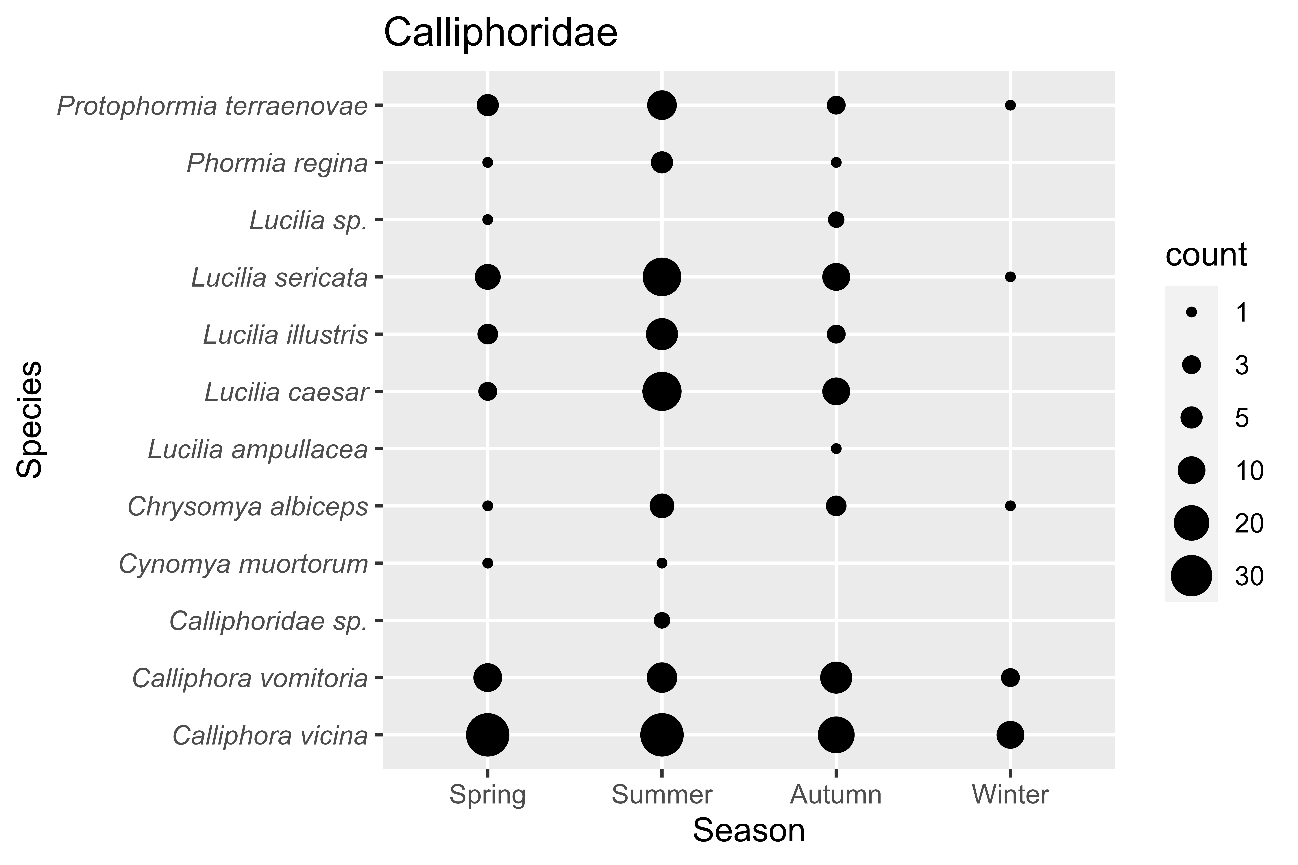

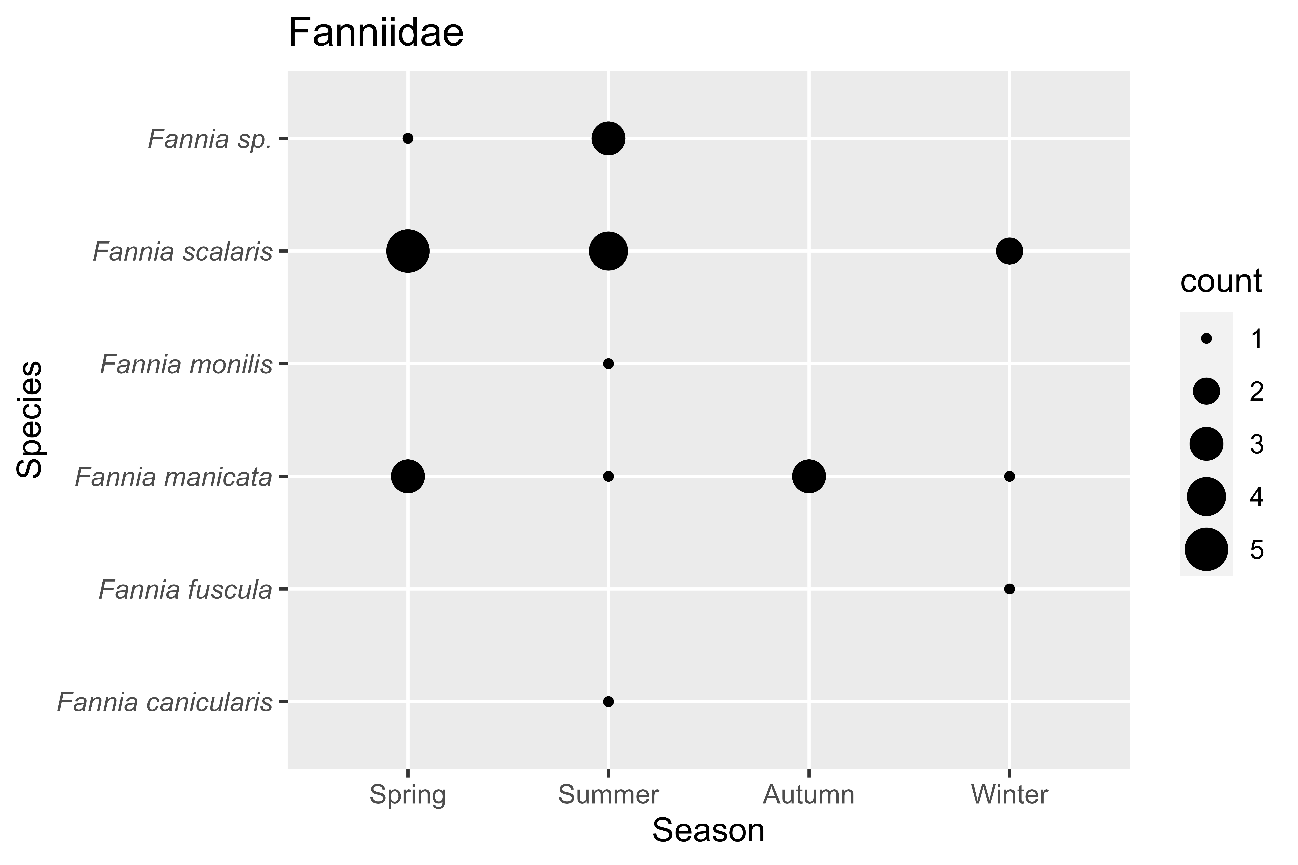

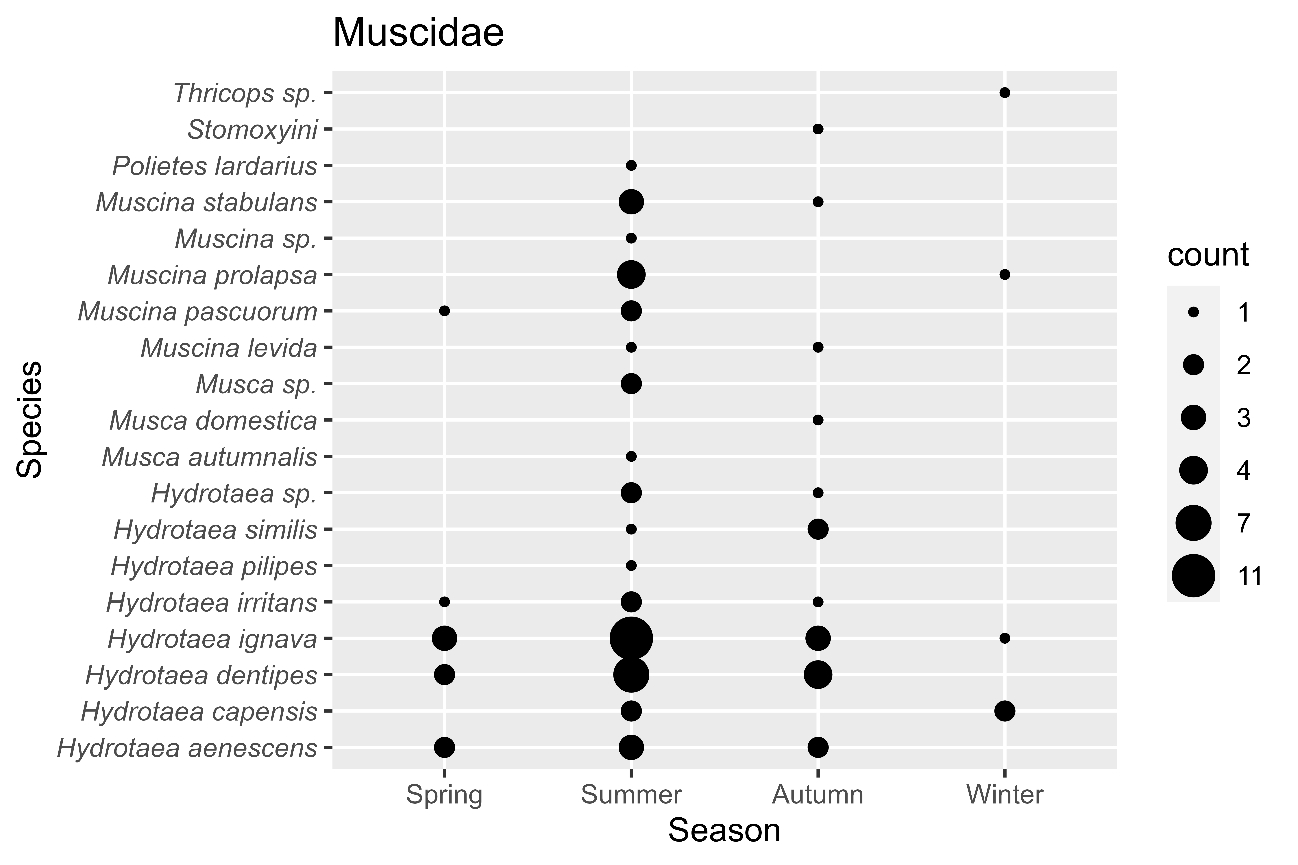

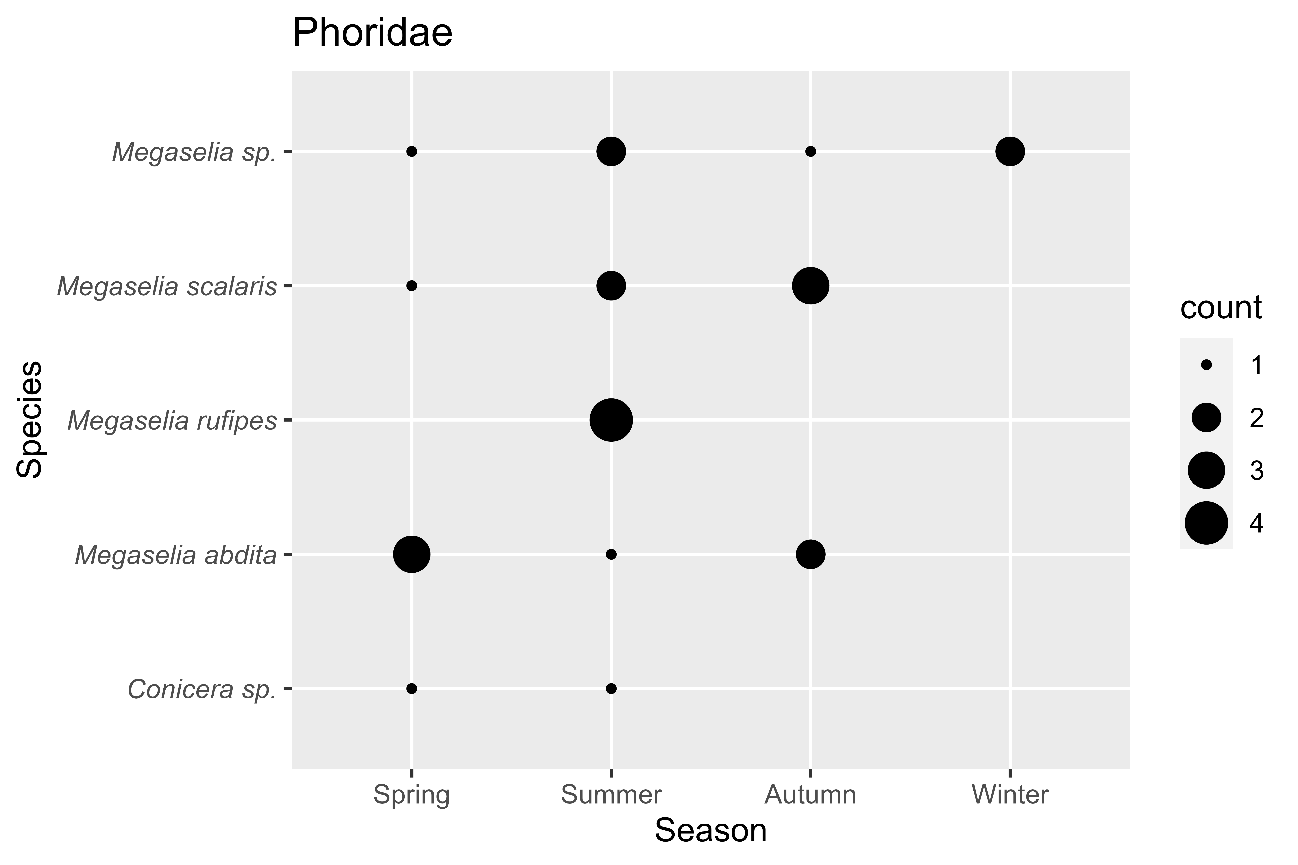

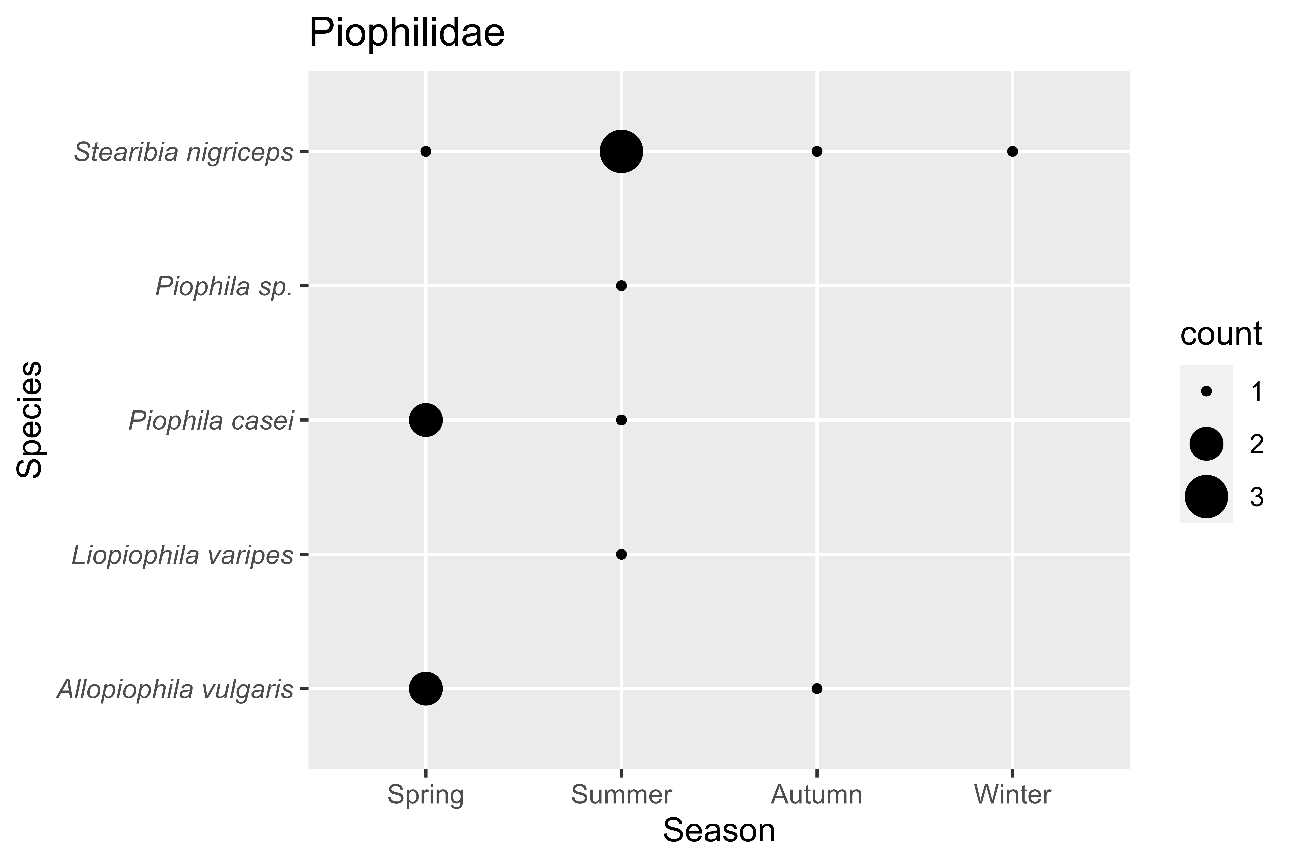

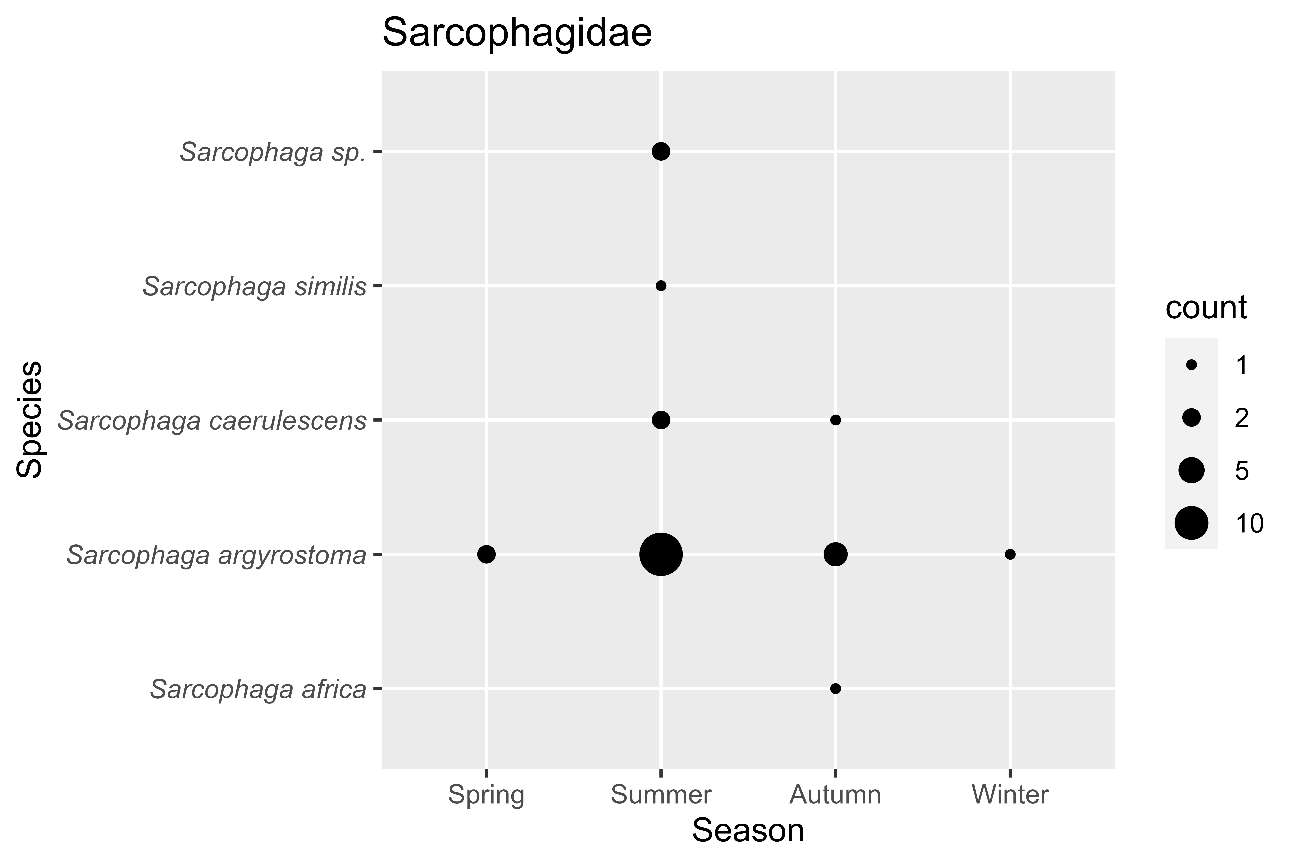

Supplement: tjad164_suppl_Supplementary_Figures [file tjad164_suppl_supplementary_figures.docx]
